# Predictive Accuracy of the Laboratory Risk Indicator for Necrotizing Fasciitis (LRINEC) Score in Forecasting Mortality and Morbidity Among Necrotizing Fasciitis Patients at a Tertiary Care Hospital in Islamabad

**DOI:** 10.7759/cureus.90126

**Published:** 2025-08-14

**Authors:** Muhammad Arsalan Ali, Usama Bin Azmat, Hamad Ali Shah, Syed Munim Hussain, Syed M Qasim, Gulfam Jameel, Aamir Ghazanfar, Muhammad Asim Mehmood

**Affiliations:** 1 General Surgery, Kahuta Research Laboratories (KRL) Hospital, Islamabad, PAK; 2 Orthopedic Surgery, Kahuta Research Laboratories (KRL) Hospital, Islamabad, PAK; 3 Urology, Kahuta Research Laboratories (KRL) Hospital, Islamabad, PAK; 4 Surgery, Kahuta Research Laboratories (KRL) Hospital, Islamabad, PAK

**Keywords:** infection, lrinec score, mortality, necrotizing fasciitis, sepsis

## Abstract

Background: Necrotizing fasciitis (NF) is a rapidly progressive, life-threatening soft tissue infection associated with high morbidity and mortality if not diagnosed and treated early. Due to its often subtle clinical presentation, early identification remains a diagnostic challenge.

Objective: This study aimed to determine the predictive accuracy of the Laboratory Risk Indicator for Necrotizing Fasciitis* *(LRINEC) score in forecasting disease-specific mortality and morbidity (sepsis) among patients admitted with NF.

Methodology: This is a validation study conducted at the Department of Surgery of Kahuta Research Laboratories (KRL) Hospital in Islamabad, Pakistan, from January 2022 to September 2023. A total of 95 patients suspected of NF were enrolled through consecutive sampling. On admission, LRINEC scores were calculated using six laboratory variables: hemoglobin, white blood cell count, serum sodium, serum creatinine, C-reactive protein (CRP), and random blood glucose. Patients were followed for 28 days to assess outcomes. Disease-specific mortality was defined as death within 28 days; sepsis was diagnosed using standard clinical criteria. Diagnostic accuracy of an LRINEC score >9 was determined through sensitivity, specificity, positive predictive value (PPV), and negative predictive value (NPV).

Results: Among 95 patients, 62 (65.3%) were male and 33 (34.7%) were female, with a mean age of 48.25 ± 12.19 years. An LRINEC score >9 was observed in 65 (68.4%) patients. For predicting mortality, an LRINEC score >9 demonstrated a sensitivity of 100%, a specificity of 49.1%, a PPV of 33.8%, and an NPV of 100%. For predicting sepsis, sensitivity was 34 (79.1%), specificity 41 (65.2%), PPV 52.3%, and NPV 87.2%.

Conclusion: An LRINEC score >9 is a highly sensitive tool for identifying patients at risk of mortality and sepsis due to NF. However, due to its moderate specificity, it should be used in conjunction with clinical assessment, imaging, and early surgical intervention to improve diagnostic precision and outcomes.

## Introduction

Necrotizing fasciitis (NF) is an uncommon yet aggressive and potentially fatal soft tissue infection that causes necrosis of the muscle, fascia, and subcutaneous tissue. It is marked by the rapid destruction of subcutaneous tissues and the underlying fascia, leading to widespread systemic infection and severe complications [1]. NF is an infrequent condition, occurring at a rate of approximately four cases per 100,000 individuals. However, its mortality rate is significantly high, ranging from 20% to 30%, and can escalate to nearly 100% if diagnosis and treatment are delayed [2,3]. NF typically affects the lower limbs, trunk, and perineum and is caused by a variety of microorganisms, including aerobic and anaerobic bacteria, group A β-hemolytic streptococci, *Vibrio* species, *Aeromonas*, and certain fungi [4,5]. Conditions that increase the risk of developing NF include diabetes mellitus, older age, atherosclerosis, alcoholism, liver cirrhosis, heart failure, chronic kidney disease, obesity, and immunosuppression [6,7]. Hematological abnormalities in NF resemble those observed in sepsis patients. These include leukocytosis, leukopenia, coagulopathy, and thrombocytopenia. Anemia may occur due to hemodilution from fluid resuscitation or as a result of hemolysis [8]. Disseminated intravascular coagulation frequently occurs in cases of severe sepsis. Elevated serum creatinine levels may indicate the presence of myositis, myonecrosis, circulating toxins, or tissue ischemia [9]. Hypocalcemia indicates fat necrosis and the deposition of calcium within necrotic tissues. Elevated C-reactive protein (CRP) levels result from bacterial infection, inflammation, and tissue necrosis. Similar to severe sepsis, patients may experience impaired renal function, hypoalbuminemia, hyponatremia, abnormal liver function, metabolic acidosis, and increased serum lactate levels [10]. Early diagnosis and prompt surgical intervention are critical to halting the rapid progression of the infection and improving patient outcomes [11]. The diagnosis is mainly made through physical examination, with additional support from laboratory tests and computed tomography (CT) imaging [12]. Successful treatment relies on a combination of thorough surgical debridement, broad-spectrum antibiotic therapy, and a multidisciplinary approach that includes intensive care, active fluid management, and effective control of sepsis [13-15]. Since not all patients recover with a single debridement, it is advisable to continue re-evaluations at intervals of 6-48 hours until no further necrosis is visible and all infected tissue has been removed. Furthermore, precise monitoring of patient physiology and serial white blood cell count should be carried out every 6-12 hours, as patients often develop organ failure, for which replacement therapy is necessary. The score could support early diagnosis and differentiation from other severe soft tissue infections that require differentiated treatment.

Objective

This study aimed to determine the predictive accuracy of the Laboratory Risk Indicator for Necrotizing Fasciitis (LRINEC) score in forecasting disease-specific mortality and morbidity (sepsis) among patients admitted with NF.

## Materials and methods

This study is a validation study conducted at the Department of Surgery of Kahuta Research Laboratories (KRL) Hospital in Islamabad, Pakistan, from January 2022 to September 2023, following the approval by the hospital's Institutional Ethical Review Committee (approval number: KRL/22-0981). A total of 95 patients were included in the study, with the sample size calculated using a cut-off LRINEC score of 9, a sensitivity of 72.7%, and a specificity of 82.1%. Mortality in NF was estimated to be 40.38%, with a margin of error of 10% for sensitivity and specificity and a confidence interval of 95%. A non-probability consecutive sampling technique was employed. Patients aged 20-70 years of either gender presenting with NF, as defined in the operational definition, were included. Exclusion criteria included patients with breast abscess, those intubated and transferred from other healthcare facilities, and patients with a history of admission or surgery in the last three months. Additionally, patients with a history of malignancy, renal impairment, chronic obstructive pulmonary disease, asthma, congestive heart failure, myocardial infarction, or chronic liver disease, as well as pregnant patients confirmed by dating scans, were excluded.

Data collection

Consenting patients who met the inclusion criteria were enrolled in the study, and ethical approval was obtained from the institutional ethical review committee. Informed consent was secured from all participants for inclusion in the research and for using their data. Demographic information and a brief clinical history were recorded. Patients were assessed at admission by the researcher under the supervision of a consultant with over 10 years of experience. Blood samples (5-10 mL) were collected to calculate the LRINEC score, based on the following parameters: hemoglobin, white blood cell count, serum sodium, serum creatinine, serum CRP, and random blood sugar levels. The LRINEC score was then calculated using the original scoring system proposed by Wong et al., where each lab parameter is assigned a weighted score and a total score ≥6 suggests a moderate risk, while ≥9 indicates a high risk for NF. Patients were followed for 28 days to monitor for mortality and sepsis, as defined in the operational definitions [12]. Study variables were documented in a proforma designed for this research (see Appendices).

Data analysis

Data were analyzed using IBM SPSS Statistics for Windows, V. 20.0 (IBM Corp., Armonk, NY, USA). Continuous variables such as age were summarized as mean ± SD for normally distributed data or median (interquartile range (IQR)) for non-normally distributed data. Categorical variables, including gender and residence status, were presented as frequencies and percentages. Sensitivity, specificity, positive predictive value (PPV), and negative predictive value (NPV) for an LRINEC score ≥9 were calculated for mortality. Stratification based on age, gender, and residence status was performed to control for effect modifiers and assess their impact on mortality and sepsis.

## Results

A total of 95 patients presenting with NF who met the inclusion criteria were enrolled in the study. The mean age was 48.25 ± 12.19 years. Out of 95, 42 (44.2%) patients belonged to the age group of 20-45 years, and 53 (55.8%) belonged to the age group of 46-70 years. Among the patients, 62 (65.3%) were male and 33 (34.7%) were female. Overall, 34 (35.8%) patients ended up as mortalities, and 72 (75.8%) had sepsis (Table 1). 

**Table 1:** Characteristics of the patients LRINEC: Laboratory Risk Indicator for Necrotizing Fasciitis

Demography	Number	Percentage
Age		
20-45 years	42	44.2%
46-70 years	53	55.8%
Gender		
Male	62	65.3%
Female	33	34.7%
LRINEC (≥9)		
Yes	65	68.4%
No	30	31.6%
Mortality		
Yes	34	35.8%
No	61	64.2%
Sepsis		
Yes	72	75.8%
No	23	24.2%

**Figure 1:**
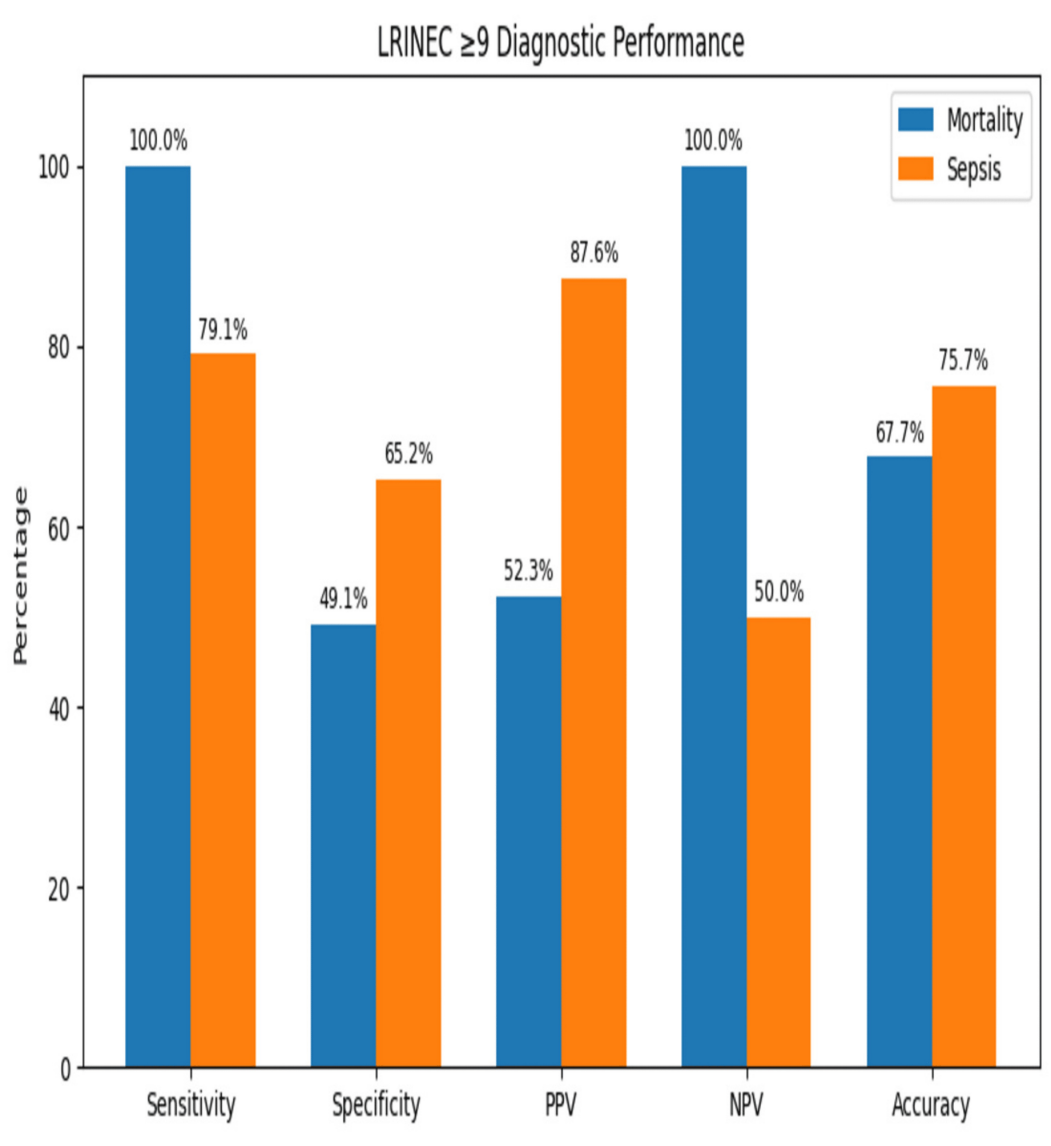
True positive, false positive, false negative, and true negative counts for LRINEC ≥9 in predicting sepsis LRINEC: Laboratory Risk Indicator for Necrotizing Fasciitis; PPV: positive predictive value; NPV: negative predictive value

Assessing the LRINEC scoring system >9 for mortality, we found that sensitivity was 100%, specificity was 49.1%, PPV was 52.3%, NPV was 100%, and predictive accuracy (DA) was 67.7%. In contrast, assessing the LRINEC scoring system >9 for sepsis, it was found that sensitivity was 79.1%, specificity was 65.2%, PPV was 87.6%, NPV was 50%, and DA was 75.7% (Table 2, Figure 1).

**Table 2:** Diagnostic performance of LRINEC score ≥9 for mortality and sepsis True positive, false positive, false negative, and true negative counts for LRINEC ≥9 in predicting sepsis. LRINEC: Laboratory Risk Indicator for Necrotizing Fasciitis; PPV: positive predictive value; NPV: negative predictive value

Outcome	Sensitivity (%)	Specificity (%)	PPV (%)	NPV (%)	Accuracy (%)
Mortality	100.0	49.1	52.3	100.0	67.7
Sepsis	79.1	65.2	87.6	50.0	75.7

## Discussion

Using an LRINEC score >9 to predict mortality, the model correctly identified 34 true positives and 30 true negatives but also misclassified 31 false positives. No false negatives were recorded. For sepsis prediction, the LRINEC score identified 57 true positives, but there were 15 false negatives, eight false positives, and 15 true negatives. The predictive performance of LRINEC score >9 for mortality showed 100% sensitivity but only 49.1% specificity, indicating it effectively identifies at-risk patients but has a high false positive rate. The PPV was 52.3%, meaning just over half of the patients classified as high-risk died, while the NPV was 100%, confirming that all patients with a low LRINEC score survived. The overall diagnostic accuracy was 67.7%. For sepsis prediction, the LRINEC score demonstrated 79.1% sensitivity and 65.2% specificity, with a PPV of 87.6% and an NPV of 50%. The overall diagnostic accuracy was 75.7%, showing a more balanced performance compared to mortality prediction.

These findings suggest that while an LRINEC score >9 effectively flags high-risk cases, its moderate specificity leads to a higher rate of false positives, which may result in unnecessary interventions. This underscores the need for clinical correlation, imaging, and additional diagnostic tools to ensure accurate risk assessment. NF is a severe, fast-progressing infection that demands early detection and prompt treatment to reduce mortality and complications. The LRINEC score is widely used to assess risk, but its accuracy has been debated. Our study evaluated its performance in predicting mortality and sepsis, revealing high sensitivity but moderate specificity, making it a useful but imperfect tool. Our findings support Wong et al., who initially introduced LRINEC as a way to distinguish NF from other soft tissue infections​. In our study, a score >9 demonstrated 100% sensitivity for mortality and 79.1% for sepsis, indicating its reliability in identifying high-risk patients [12].

However, its moderate specificity (49.1% for mortality and 65.2% for sepsis) raises concerns about false positives, potentially leading to unnecessary interventions. Ramesh et al. validated LRINEC in a tertiary care setting, highlighting its strong correlation with elevated CRP, leukocytosis, hyponatremia, and creatinine levels, all of which we observed in our study​ [16]. These findings reinforce that LRINEC is a valuable screening tool, but clinical judgment and additional diagnostics, such as imaging, remain necessary. Delayed surgical intervention in NF significantly increases mortality. Voros et al. emphasized the critical role of early, aggressive debridement, a principle our findings support, as high-risk patients (LRINEC >9) were more likely to require surgical intervention​ [17]. Similarly, Rea and Wyrick [18] noted that late recognition is a major cause of poor outcomes, which LRINEC aims to mitigate by prompting early suspicion. Beyond surgery, Majeski and Alexander stressed the importance of nutritional support and metabolic stabilization, which are essential for improving survival​ [19]. These studies collectively reinforce that successful NF management requires a combination of early diagnosis, immediate surgical debridement, and supportive care.

Despite its benefits, LRINEC has limitations. Wall et al. developed objective criteria to differentiate NF from non-necrotizing infections, arguing that clinical findings like pain disproportionate to appearance, rapid progression, and imaging should supplement laboratory markers​ [20,21]. While an LRINEC score >9 effectively flags high-risk NF cases, it is not definitive. To optimize patient management, clinicians should use LRINEC as an initial screening tool, not a stand-alone diagnostic measure; rely on imaging (MRI, CT) and bedside assessments to confirm NF; prioritize early surgical evaluation for high-risk patients; consider additional biomarkers (e.g., procalcitonin, lactate) to enhance specificity; and ensure nutritional and metabolic support alongside surgical intervention.

Limitations

This study highlights LRINEC's strengths and weaknesses. However, the moderate specificity means some patients without NF may still score high, potentially leading to overtreatment. One of the main limitations of this study is its cross-sectional design, which limits the ability to establish causal relationships between the LRINEC score and patient outcomes. The sample size of 95 patients, while sufficient for preliminary analysis, may not provide enough statistical power to detect smaller differences or outcomes. Furthermore, the study relied on the LRINEC score alone to predict mortality and morbidity, without considering other potential confounding factors, such as comorbid conditions, the timing of treatment, or the severity of infection.

## Conclusions

Based on the findings of this study, an LRINEC score >9 demonstrated strong predictive value for both sepsis and 28-day disease-specific mortality in patients with NF, with particularly high sensitivity but moderate specificity. While the score effectively identified high-risk patients, its limited ability to distinguish non-lethal cases underscores the need for cautious interpretation. The absence of false negatives for mortality reinforces the LRINEC score's utility in flagging critical cases early, yet the number of false positives indicates that it should not be used in isolation. Integrating the LRINEC score with detailed clinical examination, imaging findings, and timely surgical intervention remains essential for accurate diagnosis and improved patient outcomes. LRINEC score remains a valuable tool for early risk stratification; further research involving larger, multi-center cohorts is warranted to validate its performance and explore the integration of additional biomarkers or imaging criteria to improve specificity and overall diagnostic accuracy.

## Appendices

**Table 3:** Data collection proforma LRINEC: Laboratory Risk Indicator for Necrotizing Fasciitis

Section	Variable	Option/response
Section 1: Patient information	Patient ID	
	Age	20-45 years
		46-70 years
		>70 years
	Gender	Male
		Female
	Clinical diagnosis	Necrotizing fasciitis
		Others (specify):
Section 2: Laboratory parameters (LRINEC score)	Serum sodium (mmol/L)	
	Serum potassium (mmol/L)	
	Serum creatinine (mg/dL)	
	White blood cell count (×10³/mm³)	
	Hemoglobin level (g/dL)	
	C-reactive protein (CRP) (mg/L)	
Section 3: Clinical outcomes	Mortality	Yes
		No
	Sepsis	Yes
		No
	Treatment given	Surgical intervention
		Antibiotics
		Others (specify):
	Outcome	Recovery
		Complications (specify):
	Follow-up duration (days)	
	Patient status	Stable
		Improved
		Deteriorated
	Complications (if any)	Yes
		No
	If Yes, specify complications	
Section 4: Additional observations	Other observations	
